# Impact of a digital mindfulness and motor imagery intervention on pain and perceived function in 203 patients with musculoskeletal pain

**DOI:** 10.3389/fdgth.2026.1895131

**Published:** 2026-09-21

**Authors:** Guillaume Zunzarren, Bertrand Garet, Sonja Cabarkapa, Jérôme Murgier

**Affiliations:** 1 Orthopedic Department, Aguiléra Private clinic, Ramsay Santé, Biarritz, France; 2 St Vincent's Hospital, Melbourne, VIC, Australia

**Keywords:** central activation dysfunction, chronic pain, digital therapeutics, mindfulness, motor imagery, musculoskeletal pain, neuromuscular control, real-world evidence

## Abstract

**Background:**

Musculoskeletal pain (MSP) is associated with persistent pain, functional impairment, and altered neuromuscular control mechanisms. Mindfulness and motor imagery interventions may target central sensorimotor dysfunctions involved in pain persistence. This study evaluated the effects of a digital intervention combining mindfulness and guided motor imagery on pain and perceived function in patients with MSP.

**Methods:**

This observational study analyzed 203 consecutive users with complete datasets who used a standardized digital intervention delivered through the Yuzit® application. The program combined mindfulness exercises and guided motor imagery sessions adapted to the painful anatomical area, covering eight musculoskeletal regions. Outcomes were assessed at baseline (T0), before the third session (T1), and after program completion (T2). Pain intensity was measured using the 11 points Numerical Rating Scale (NRS; 0–10) and perceived function using the Single Assessment Numeric Evaluation (SANE). The Minimal Clinically Important Difference (MCID) was defined as a VAS reduction ≥ 2 points. Secondary analyses evaluated the influence of age, sex, symptom duration, pain location, and baseline pain severity.

**Results:**

The cohort included 122 women (60.1%) and 81 men (39.9%) with a mean age of 46.4 ± 16.6 years. Mean NRS improved from 5.17 ± 2.00 at T0 to 3.26 ± 2.40 at T2 (*Δ* = −1.91; t = −12.45; *p* < 0.000001), while mean SANE improved from 54.7 to 65.3 (*Δ* =  + 10.5 points; +19.2%). The MCID was achieved by 50.2% of patients, and 74.4% showed any improvement. Improvement was observed across all anatomical locations and symptom durations. In patients with chronic symptoms lasting more than one year (*n* = 117; 57.6%), 74.4% of patients improved and 51.3% achieved the MCID (t = −9.56; *p* < 0.001), with no significant difference across chronicity groups (one-way ANOVA: F = 0.485; *p* = 0.616). Patients with higher baseline pain showed greater improvements (NRS ≥ 6: *Δ*NRS = −2.53; *Δ*SANE =  + 14.1). Satisfaction was high, with 64.0% of patients reporting satisfaction scores ≥ 4/5.

**Conclusions:**

A standardized digital intervention combining mindfulness and guided motor imagery was associated with rapid and clinically relevant improvements in pain and perceived function across all musculoskeletal regions and patient profiles, including chronic pain populations. Randomized controlled studies with objective outcomes are required to confirm these findings and investigate potential mechanisms.

## Introduction

Musculoskeletal pain (MSP) is one of the leading causes of functional disability and reduced quality of life in the general population (1). Its high prevalence and tendency to become a chronic condition make it a major public health issue (2). Management generally relies on a mixed therapeutic approach combining medication (3) with non-pharmacological therapies such as rehabilitation (4, 5). However, some patients' pain becomes chronic, which raises the issue of long-term side effects of medication (6) as well as increased morbidity and loss of function (7). A growing body of research suggests that central mechanisms, involving pain modulation, body image, and neuromuscular control, play a crucial role in the progression of MSP, particularly in its chronic or treatment-resistant forms (8–10). In this context, altered neuromuscular activation appears to be a key element. Muscle activation deficits can persist independently of any active peripheral involvement, reflecting dysfunction of central sensorimotor circuits. This perspective leads us to move beyond strictly peripheral models to integrate a central dimension of motor and pain disorders (11).

Approaches based on motor imagery and mindfulness aim to act on these central mechanisms (12). Motor imagery engages neural processes involved in movement planning and representation without requiring physical execution, while mindfulness-based approaches may influence attention, body awareness, interoception, and the experience of pain. Recent controlled work in fibromyalgia has reported changes in pain and interoceptive awareness after a brief mindfulness self-compassion intervention (13). The combination of mindfulness and motor imagery may therefore be relevant when pain, fear of movement, or impaired motor confidence limits physical engagement; however, their respective contributions cannot be separated when delivered as a combined intervention. Central Activation Dysfunction (CAD) (14) has been proposed to describe a central alteration of neuromuscular activation that may contribute to the persistence of pain and functional deficits, independently of any objectively observable peripheral involvement. This phenomenon is particularly visible after ACL reconstruction surgery and is the subject of much current research (10, 15–17).

Interventions delivered using digital tools offer a particularly suitable framework for exploring these mechanisms (18–20). They allow for complete standardization of protocols, independent of the therapist, guaranteeing high reproducibility of intervention and evaluation conditions. This characteristic is essential for the study of central phenomena, the analysis of which requires homogeneous and rigorously controlled methods. In this study, this approach was implemented via a digital application, Yuzit®, specifically designed to translate the principles of CAD, mindfulness, and guided motor imagery into an operational and standardized way (21).

The primary objective of this study was to evaluate the impact of a digital program combining mindfulness and motor imagery on pain and perceived function in patients with musculoskeletal pain regardless of symptom duration and location. Secondary objectives were to explore the influence of individual factors such as age, sex, and body mass index on clinical response, and to assess the acceptability of the intervention through patient satisfaction levels.

We hypothesized that participants would report reduced pain and improved perceived function during use of the standardized digital intervention. A secondary, mechanistic hypothesis was that central sensorimotor processes could contribute to these changes; because no neurophysiological or objective neuromuscular measurements were obtained, this mechanism was not tested directly.

## Materials and methods

### Study design

This observational study evaluated the effects of a digital intervention combining mindfulness and guided motor imagery in patients with musculoskeletal pain. The study analyzed prospectively collected anonymized data from consecutive users of the Yuzit® application. The primary objective was to assess changes in pain and perceived function during the intervention period.

### Participants

The study included all consecutive users who completed the full programme within the application. Completion of the programme required responding to the three assessments (T0, T1, T2). Inclusion criteria were: age ≥18 years and presence of musculoskeletal pain affecting any anatomical region. A total of 203 consecutive users completed the programme and were included in the analysis.

### Digital intervention

The intervention was delivered exclusively through the Yuzit® application and consisted of sessions combining mindfulness and guided motor imagery exercises adapted to the painful anatomical area.

Each session included two sequential phases:

1. Mindfulness phase: a 2–3-minute guided attentional exercise focused on breathing, body awareness, and muscle perception, aiming to reduce cognitive agitation and promote attentional readiness.
2. Motor imagery phase: patients observed a short video demonstrating a targeted movement and subsequently performed guided mental visualization of the same movement without physical execution. Verbal guidance emphasized muscle contraction sensation, movement fluidity, and internal perception of movement.

Sessions lasted between 10 and 50 min depending on the anatomical area. Patients were instructed to perform at least two sessions per week, separated by 48–72 h. Programs generally consisted of 5–6 sessions per anatomical area.

### Outcome measures

Assessments were performed at three predefined time points:

- T0: baseline before any intervention;
- T1: before the third session;
- T2: after completion of the program.

Pain intensity was recorded within the application on a 0–10 numerical scale and is therefore reported as an 11-point Numerical Rating Scale (NRS), where 0 indicated no pain and 10 the highest pain rating. Perceived physical function was assessed using the Single Assessment Numeric Evaluation (SANE; 0–100), a single-item patient-reported measure with reported concurrent validity across several musculoskeletal regions (22). In this heterogeneous multi-region cohort, SANE was used as a pragmatic global measure of perceived function rather than as a region-specific validated instrument. The threshold SANE ≥80 was used only as a descriptive benchmark and was not treated as a validated clinically important threshold. Patient satisfaction was recorded at T1 and T2 using a 5-point Likert scale.

Demographic and clinical variables collected included age, sex, body mass index (BMI), pain location, symptom duration, context of onset, medication use, and previous treatments.

### Ethical considerations

This observational study was conducted in accordance with the principles of the Declaration of Helsinki. All participants provided informed consent prior to participation. The analyzed data originated exclusively from self-administered questionnaires completed within the Yuzit® application. All data were fully anonymized prior to extraction and analysis, with no access to personally identifiable information by the investigators. Data were securely hosted on servers located in France (OVH) in compliance with the General Data Protection Regulation (GDPR). The study was strictly non-interventional and involved no modification of patient management or standard care.

Due to the retrospective analysis of fully anonymized data and the absence of any possibility of patient re-identification, formal ethics committee approval was not required under applicable French regulations. The study consisted of a retrospective secondary analysis of previously collected, irreversibly anonymized data. On this basis, the authors considered the analysis outside the scope of research requiring prospective review by a French Comité de Protection des Personnes (CPP).

### Statistical analysis

Descriptive statistics were used to summarize demographic and clinical characteristics. Continuous variables are presented as means ± standard deviations, and categorical variables as frequencies and percentages. Changes in NRS and SANE scores between time points were analyzed using paired two-tailed Student's t-tests. For the principal T0-to-T2 VAS change, the mean difference is reported with a 95% confidence interval and standardized paired effect size (Cohen dz). Between-group comparisons were performed using independent-samples Student's t-test or one-way ANOVA. Associations between continuous variables were assessed using Pearson's correlation coefficient. Statistical significance was set at *α* = 0.05. All analyses were performed using Python 3 (Pandas, SciPy). Subgroup analyses according to age, sex, symptom duration, anatomical location, pain origin, and baseline pain severity were exploratory; no adjustment for multiple comparisons was applied, and these findings should therefore be interpreted cautiously. Because the analytic cohort comprised complete cases at all three time points, a mixed-effects model was not required for missing-data handling; the paired analyses were retained to address the prespecified within-participant contrasts.

## Results

### Study population

A total of 203 consecutive users were included in the analysis. The population comprised 122 women (60.1%) and 81 men (39.9%). Mean age was 46.4 ± 16.6 years (range 18–82). Mean BMI was 24.1 kg/m^2^. The majority of patients presented with symptoms lasting more than one year (*n* = 117, 57.6%). Baseline characteristics are summarized in Table 1.

**Table 1:** Baseline demographic and clinical characteristics (*N* = 203).

Chateristic	Value	Detail
Total sample	203	—
Age, mean ± SD (range)	46.4 ± 16.6 years	18–82
Female sex, *n* (%)	122 (60.1%)	81 men (39.9%)
BMI, mean (kg/m^2^)	24.1	Individual calculation
Program duration, median (mean ± SD)	22.5 days	28.0 ± 22.7 days
Knee	67 (33.0%)	Most frequent zone
Hip/Pelvis	52 (25.6%)	—
Ankle/Foot	27 (13.3%)	—
Shoulder	18 (8.9%)	—
Lumbar spine	18 (8.9%)	—
Other zones	21 (10.3%)	Elbow, neck, wrist/hand
Chronic symptoms > 1 year	117 (57.6%)	Largest subgroup
Subacute symptoms 3–12 months	57 (28.1%)	—
Acute symptoms < 3 months	29 (14.3%)	—
Sports-related pain origin	57 (28.1%)	Traumatic: 48 (23.6%)
Functional users (VAS T0 = 0)	5 (2.5%)	No pain at baseline

### Changes in pain (VAS)

Mean NRS scores were 5.17 ± 2.00 at baseline (T0), 4.04 ± 2.43 at T1, and 3.26 ± 2.40 at T2. The mean change was −1.13 points between T0 and T1 and −1.91 points between T0 and T2. For the T0-to-T2 paired comparison, the mean difference was −1.91 points (95% CI −2.21 to −1.61; Cohen dz = 0.87; t = −12.45; *p* < .001). Clinically, 74.4% of patients showed any improvement (*Δ*NRS < 0), 50.2% achieved the Minimal Clinically Important Difference (MCID, defined as a reduction of ≥ 2 points on the 0–10 NRS scale 23), and 19.2% showed an improvement of ≥ 4 points. At T2, 31 patients (15.3%) reported complete absence of pain (NRS = 0), of whom 28 had reported pain at baseline, corresponding to complete pain resolution in 14.1% of patients with baseline pain. Results are presented in Tables 2, 3.

**Table 2:** NRS score (0–10) at three time points (*N* = 203).

Time point	Mean ± SD	Median	ΔNRS	*p*-value
T0 — Baseline	5.17 ± 2.00	5.0	—	Ref.
T1 — Mid-program	4.04 ± 2.43	4.0	−1.13	< 0.001
T2 — End of program	3.26 ± 2.40	3.0	−1.91	< 0.000001

Paired two-tailed Student's t-test versus T0. t = −12.45 at T2.

Bold values indicate the main findings highlighted in each table.

**Table 3:** Distribution of NRS response categories at T1 and T2 (*N* = 203). ≥ 2-pt resp. = MCID (minimal clinically important difference, ΔNRS ≤ −2 [34,35])*.*

Response category	N T1	% T1	N T2	% T2
Any improvement (ΔNRS < 0)	108	53.2	**151**	**74**.**4**
of which: large improvement (ΔNRS ≤ −4)	—	—	39	19.2
of which: complete resolution (NRS T2 = 0)	—	—	28	14.1%
No change (*Δ*NRS = 0)	81	39.9	42	20.7
Worsening (*Δ*NRS > 0)	14	6.9	10	4.9
MCID achieved (≥ 2-pt resp.)	59	29.1	**102**	**50**.**2**

Bold values indicate the main findings highlighted in each table.

### Changes in perceived physical function (SANE)

Mean SANE scores were 54.7 ± 19.4 at T0, 58.8 ± 19.9 at T1, and 65.3 ± 21.9 at T2. The mean improvement was +4.1 points between T0 and T1 and +10.5 points between T0 and T2 (+19.2%). Clinically, 56.2% of patients showed concordant improvement in both VAS and SANE. The proportion of patients achieving a SANE score ≥ 80 increased from 10.8% at T0 to 42.4% at T2 (Table 4).

**Table 4:** SANE score (0–100; higher = better perceived function) at three time points (*N* = 203). Total gain T0→T2: +10.5 points (+19.2%)*.*

Time point	Mean ± SD	Median	ΔSANE	*p*
T0	54.7 ± 19.4	53.0	—	Ref.
T1	58.8 ± 19.9	60.0	+4.1	< 0.001
T2	65.3 ± 21.9	70.0	**+10.5**	**< 0.001**

Bold values indicate the main findings highlighted in each table.

### Satisfaction (secondary endpoint)

The mean satisfaction score at T2 was 3.87 ± 0.98 out of 5. Overall, 64.0% of patients reported a score ≥ 4 (satisfied or very satisfied). Satisfaction improved between T1 and T2 in 36.9% of users, remained stable in 49.3%, and decreased in 13.8%. Notably, even users reporting dissatisfaction (score 2) at T2 showed a mean VAS reduction of 2.00 points (69% responders), indicating that clinical efficacy and subjective satisfaction are partly independent dimensions (Table 5).

**Table 5:** Patient satisfaction at T1 and T2 (*N* = 203), with mean ΔNRS and responder rate per satisfaction level at T2*.*

Satisfaction level	N T1	% T1	N T2	% T2	ΔNRS T2	Resp. T2
1 — Very dissatisfied	9	4.4	6	3.0	—	—
2 — Dissatisfied	25	12.3	18	8.9	−2.00	69%
3 — Neutral	57	28.1	49	24.1	−1.72	68%
4 — Satisfied	51	25.1	58	28.6	−1.76	76%
5 — Very satisfied	61	30.0	**72**	**35**.**5**	−2.31	80%
Mean score/5	**3.61**	—	**3.87**	—	—	—
Highly satisfied (4–5)	112 (55.2%)		**130 (64.0%)**		—	—

Bold values indicate the main findings highlighted in each table.

### Sub-group analyses

#### Anatomical location

Improvement was observed across all anatomical regions (Table 6). The largest observed reductions were observed for the lumbar spine (*Δ*NRS = −2.39; 89% responders) and ankle/foot (ΔNRS = −2.30; ΔSANE = + 15.6). The knee program, the most represented location (*n* = 67, 33.0%), showed a more moderate response (*Δ*NRS = −1.75; ≥ 2-pt resp. 49%).

**Table 6:** Results by anatomical zone, ranked by ΔNRS.

Anatomical zone	*N*	NRS T0	NRS T2	ΔNRS	Resp.	≥ 2-pt resp.	ΔSANE
Elbow	5	4.80	2.40	−2.40	80%	40%	+6.5
Lumbar spine^a^	18	5.67	3.28	**−2**.**39**	**89%**	**56%**	+9.6
Ankle/Foot^a^	27	5.52	3.22	−2.30	**74%**	59%	+15.6
Hip/Pelvis	52	4.92	2.96	−1.96	81%	54%	+14.0
Shoulder	18	5.06	3.12	−1.94	72%	44%	+8.4
Knee^b^	67	5.09	3.34	−1.75	70%	49%	+8.8
Cervical/Neck^c^	7	5.71	4.71	−1.00	71%	29%	+4.0
Wrist/Hand/Finger^c^	9	4.33	3.22	−1.11	44%	33%	+9.2
TOTAL	**203**	**5**.**17**	**3**.**26**	**−1**.**91**	**74.4%**	**50.2%**	**+10**.**5**

a Above cohort average.

b Below cohort average.

c Insufficient sample (*n* < 10). ≥ 2-pt resp. = proportion with ΔVNRS ≤ −2 (MCID [34,35]).

Bold values indicate the main findings highlighted in each table.

#### Duration of symptoms

Improvement was observed regardless of symptom duration. In patients with symptoms lasting more than one year (*n* = 117), *Δ*NRS = −1.86 and *Δ*SANE =  + 8.7, with 74.4% of responders and 51.3% achieving MCID. One-way ANOVA across the three chronicity groups (acute, subacute, chronic) was non-significant (F = 0.485; *p* = 0.616). In the chronic severe subgroup (NRS T0 ≥ 6; *n* = 64), ΔVAS reached −2.11 with 54.7% achieving MCID (Table 7).

**Table 7:** Results by symptom duration.

Group	*N*	NRS T0	NRS T2	*Δ*NRS	Resp.	≥ 2-pt resp.	Sat. T2
Acute (< 3 months)	29	5.34	3.10	−2.24	79.3%	55.2%	4.21
Subacute (3–12 months)	57	4.53	2.82	−1.70	68.4%	40.4%	3.79
Chronic (> 1 year)	**117**	5.41	3.55	**−1**.**86**	**74**.**4%**	51.3%	3.84
Chronic severe (> 1 yr + VAS ≥ 6)	64	≥ 6.0	—	−2.11	73.4%	54.7%	3.75

ANOVA: F = 0.485; *p* = 0.616 (*N*S). Paired t-test vs. 0 for chronic subgroup: t = −9.56; *p* < 0.001. ΔSANE: acute +13.3; subacute +14.1; chronic +8.7*.*

Bold values indicate the main findings highlighted in each table.

#### Sex

The male population showed better response than women with a statistically significant difference between groups (t = −3.95; *p* < 0.001). (Table 8).

**Table 8:** Results by sex (*N* = 203).

Group	N (%)	NRS T0	ΔVAS	Resp.	≥ 2-pt resp.	ΔSANE
Male	81 (39.9%)	—	**−2.63**	**81%**	**58%**	+13.6
Female	122 (60.1%)	—	−1.43	70%	45%	+8.8
t = −3.95 · *p* = 0.0001	—	—	—	—	—	—

Independent samples two-tailed Studen*t's t-test.*

Bold values indicate the main findings highlighted in each table.

#### Age and BMI

Improvement was observed across all age groups (Table 9). Patients aged ≥ 60 years showed the highest responder rate (80%; ΔNRS = −2.27; ΔSANE = + 11.2). Age was not a linear predictor of response (Pearson r = −0.071; *p* = 0.312). BMI was confirmed as a non-predictor (r = 0.003; *p* = 0.970), supporting universal accessibility of the program regardless of age or body weight.

**Table 9:** Results by age group (*N* = 203).

Age group	N	ΔNRS	Resp.	≥ 2-pt resp.	ΔSANE	Pearson r
< 30 years	39	−2.05	74%	56%	+14.2	r = −0.071
30–44 years	57	−1.37	70%	39%	+9.1	*p* = 0.312 (*N*S)
45–59 years	66	−2.08	74%	53%	+10.8	Non-predictor
≥ 60 years	41	**−2**.**27**	**80%**	56%	+11.2	BMI: r = 0.003 NS

Age and BMI are confirmed non-predictors of treatment response.

Bold values indicate the main findings highlighted in each table.

#### Initial pain intensity

Participants with higher baseline pain showed larger observed reductions NRS score. Patients with an initial NRS ≥ 6 showed a ΔNRS of −2.53 and a *Δ*SANE of +14.1, with 58.2% achieving MCID (ΔNRS ≥ 2 points).

#### Pain origin

Improvement was observed across all pain origins (Table 10). Professional origin showed the highest absolute results (ΔNRS = −3.75; 92% responders; ≥ 2-pt resp. 83%; *n* = 12), though the limited sample size precludes inferential analysis. Traumatic origin showed the lowest MCID rate (31%; *n* = 48).

**Table 10:** Results by pain origin (*N* = 203).

Pain origin	*N*	*Δ*NRS	Resp.	≥ 2-pt resp.	Note
Professional^a^	12	**−3**.**75**	**92%**	**83%**	Limited *N*
Undetermined	56	−2.34	82%	59%	Above average
Sports-related	57	−1.82	74%	54%	Within average
Mixed/Other	30	−1.40	67%	43%	Heterogeneous
Traumatic^b^	48	−1.38	67%	**31%**	Lowest MCID

a above cohort average; limited sample (*n* = 12).

b lowest responder rates.

Bold values indicate the main findings highlighted in each table.

#### Anatomical location

Improvement was observed across all anatomical regions. The most significant responses were observed for the lumbar spine (ΔNRA = −2.39; 89% responders) and ankle/foot (ΔNRS = −2.30; ΔSANE = + 15.6). The knee program, the most represented location (*n* = 67), showed a more moderate response (ΔVAS = −1.75; MCID 49%).

## Discussion

This observational study suggests that a digital intervention combining mindfulness and guided motor imagery may be associated with clinically relevant improvements in pain and perceived function in patients with chronic musculoskeletal pain. Improvements were observed across multiple anatomical regions and symptom durations, including patients with symptoms persisting for more than one year. These findings support the hypothesis that central neurocognitive mechanisms may contribute substantially to persistent musculoskeletal pain and functional impairment.

The direction of the observed changes is consistent with previous studies of mindfulness-based and motor imagery interventions in musculoskeletal and chronic pain conditions (24–27). The present intervention combined both components in a single package and therefore cannot determine whether changes were related to mindfulness, motor imagery, their combination, expectancy, engagement, concurrent care, natural symptom variation, or other factors.

However, unlike conventional rehabilitation approaches focused primarily on peripheral biomechanical factors, the present intervention specifically targeted attentional processes, body perception, and voluntary motor representation. This conceptual framework could be aligned with the proposed model of Central Activation Dysfunction (CAD) (14), which postulates that persistent pain may partly reflect altered central motor command and sensorimotor integration rather than solely peripheral tissue pathology (10, 12). Within the broader landscape of digital musculoskeletal rehabilitation, the present results are consistent with and extend the findings of the most recent network meta-analysis by Zou et al. (28) which identified self-managed rehabilitation applications as the most effective telerehabilitation modality for chronic MSK pain [SMD = −0.89 (95% CI: −1.31 to −0.48)]. A systematic review and meta-analysis by Jang et al. (29), including 54 studies and 6,240 patients, concluded that digital healthcare rehabilitation is non-inferior to conventional care for musculoskeletal pain and quality of life outcomes. In the present study, patients with higher initial VAS scores demonstrated greater reductions in pain and larger functional gains. While part of this effect may be explained by a statistical floor effect inherent to pain scales (30), the parallel improvement in SANE scores suggests that the observed response may not be purely mathematical.

The digital format may offer practical advantages, including standardized home-based delivery and a relatively low implementation burden. Its remote and flexible nature may also facilitate use across a broad range of patient profiles. These characteristics complement established pharmacological or non-pharmacological approaches such as exercise therapy, for which recent meta-analyses have reported mean pain reductions of approximately 1.5–2.0 points on a 0–10 scale (31).

This study has several limitations. First, its observational and uncontrolled design does not permit causal inference. Regression to the mean, expectancy effects, concurrent treatment, natural symptom fluctuation, and selective programme completion are plausible alternative explanations. Second, outcomes were exclusively self-reported, with no objective functional, neuromuscular, or neurophysiological measurements. Third, follow-up was short and does not establish persistence of change. Fourth, the heterogeneous anatomical regions and small size of some subgroups limit subgroup interpretation. Fifth, the subgroup analyses were exploratory and were not adjusted for multiple testing. Finally, SANE is a pragmatic single-item measure whose validity and interpretation can vary by anatomical region, and the SANE ≥80 threshold used here is descriptive rather than a validated cross-region clinical threshold.

Despite these limitations, the findings support the feasibility of evaluating a standardized digital mindfulness and motor imagery programme in routine use and provide preliminary estimates of changes in self-reported pain and perceived function among programme completers. Randomized controlled studies are needed to estimate causal treatment effects, and mechanistic studies incorporating objective motor-control and neurophysiological measures are needed to test hypotheses concerning central sensorimotor modulation.

## Conclusion

This observational study shows that a digital program combining mindfulness and motor imagery is associated with a rapid, significant, and clinically relevant improvement in pain and perceived function in patients with musculoskeletal pain, regardless of its location. The uncontrolled design precludes attribution of these changes specifically to the intervention or to a central neurophysiological mechanism. These findings should therefore be considered hypothesis-generating and warrant confirmation in randomized controlled studies with objective outcomes and longer follow-up.

## Data Availability

The raw data supporting the conclusions of this article will be made available by the authors, without undue reservation.
